# Study of Demographics, Clinical Profile and Risk Factors of Inguinal Hernia: A Public Health Problem in Elderly Males

**DOI:** 10.7759/cureus.38053

**Published:** 2023-04-24

**Authors:** Puneet K Agarwal

**Affiliations:** 1 Department of General Surgery, All India Institute of Medical Sciences, Bhopal, Bhopal, IND

**Keywords:** geriatric, constipation, heavy weight, risk factors, inguinal hernia

## Abstract

Background: Inguinal hernia repair is the most commonly performed elective surgery in India draining significant healthcare resources. This observational study was conducted at a tertiary-level institute in northern India to look into the demographics, clinical profile and risk factors of inguinal hernia.

Method: This study was conducted as an observational study at the tertiary care centre of northern India, including 110 patients who had come to the surgical outpatient department for inguinal hernia repair. After obtaining informed consent from all the participants, demographic details, history and clinical examination were recorded. This was a prospective, single-centre, non-randomized, observational study.

Results and discussion: In our study, 43 patients (39%) were >50 years of age. One hundred and seven patients (97.27%) were males, and three (2.72%) were females. Male: Female ratio was 32:1. The preponderance of males was due to their involvement in more strenuous exercises and lifting weights and the anatomical differences between them. The main risk factor in the present study was lifting heavy weights 55%, followed by altered bowel habits 36.36% and respiratory disease (chronic obstructive airway disease). Smoking and diabetes were also associated as risk factors for the hernia. In this study, the most common side of hernia was on the right side, 63%, on the left, 33% and bilateral in 4% of patients. The indirect hernia was the most common type.

Conclusion: Inguinal hernia is a surgical problem found commonly in the male elderly. Right-sided inguinal hernia is common, with the indirect type being more frequent. Heavy weight lifting and strenuous exercises were commonly found risk factors.

## Introduction

Inguinal hernia repair is the most commonly performed elective surgery in India. Hernia affects 15%-20% of the general population. The prevalence of inguinal hernia in India is estimated to be 1.5 to 2 million [1]. Inguinal hernia is most common in men than women. About 90% of inguinal hernia repairs are done in men, whereas 70% of femoral hernia repairs are performed in women. The estimated lifetime risk of inguinal hernia in men is 27% and 3% in women [2]. Prevalence of inguinal hernia is age dependent, and in males, it has a bimodal distribution curve, with the first peak in the first year of age and the second peak after the fourth decade of life [3,4]. Though femoral hernia is most common in women, overall, the most common hernia in women is an inguinal hernia, i.e. inguinal hernias are five times more common than femoral hernias.

The most common subtype of groin hernia in men and women is the indirect inguinal hernia. The ratio between indirect hernia and direct hernia in men is 2:1 [4,5].

Classical hernia classification is based on the relation between hernia and surrounding structures and is classified as indirect, direct, and femoral. Direct hernias protrude medially to the inferior epigastric vessels within Hesselbach’s triangle. And hernias lateral to inferior epigastric vessels, through the deep ring, are indirect hernias. Femoral hernias protrude through the femoral ring and are seen as a bulge lateral to the pubic tubercle. Many other hernia classifications have been proposed; the most commonly used are the Nyhus classification of groin hernia, the European hernia society groin hernia classification and Zollinger’s unified classification of groin hernias. The most common presentation of an inguinal hernia is a lump in the groin that increases on standing and decreases/disappears on lying down. Significantly few patients have associated groin pain or discomfort. Depending on the contents of the sac, the patient can have extra inguinal symptoms like altered bowel habits or urinary symptoms. Presentation in surgical emergencies includes irreducibility, intestinal obstruction and strangulation of the contents and should be intervened early. History regarding complications of hernia should be ruled out. History regarding reducibility is essential if there are symptoms of groin pain [4,5].

Inguinal hernias can present in the paediatric age group (is congenital) or later in adults, which is usually considered acquired. The persistence of the patent processus vaginalis (PPV) has been implicated in the etiopathogenesis of congenital hernia. The mere existence of PPV alone doesn’t lead to an inguinal hernia. PPV and other risk factors like family history, tissue weakness and strenuous activity predispose to inguinal hernia [5].

The aetiology of inguinal hernia in adults is multifactorial and influenced by occupational, environmental and hereditary factors. Hypothetically, obesity should have been a high-risk factor for an inguinal hernia. But studies have shown the incidence of inguinal hernia decreased in overweight and obese patients [6,7].

This observational study was conducted at a tertiary-level institute in northern India on inguinal hernia patients subjected to surgery. Their clinical profile, demographics and associated risk factors were recorded.

## Materials and methods

The study was conducted as an observational study at a tertiary care centre in northern India. All the study subjects who visited the hospital with complaints of groin swelling with or without pain were included and subjected to surgery after obtaining informed consent and demographic details were obtained.

The surgeon performed a thorough clinical examination and explained privacy and confidentiality to the patient. This was a prospective, single-centre, non-randomized, observational study. The study was approved by the Institutional Human and Ethical Committee (IHEC) of the Institute.

Inclusion criteria

All patients diagnosed with an inguinal hernia visited the surgical outpatient department.

Exclusion criteria

Patients with recurrent inguinal hernia, incarcerated hernia, physical or mental disorders, patients with impaired cognition, patients using daily analgesics for any other illness, refusal of consent to participate in the study, patients with a history of surgery in the groin area will be excluded from the study.

Patient details, such as name, age, sex, address, socio-economic status and occupation were noted down. A detailed history was taken concerning the presenting complaint and the history of the present illness. Comorbidities were noted down. Patients with pain in the groin preoperatively were noted, and their pain was assessed on a numerical rating scale of 1 to 10 using the visual analog score. Pain is graded into four categories depending upon the VAS scores as Nil = VAS score 0; Mild = VAS score 1-3; Moderate = VAS score 4-6; Severe = VAS score >6

Statistical analysis

Descriptive statistics were used, where for nominal variables, numbers and percentages were used, while for numerical variables, the mean was calculated. The association between two nominal variables was tested by using the chi-square test. A p-value less than 0.05 was considered statistically significant.

## Results

One hundred and ten patients who visited the surgical outpatient department for inguinal hernia repair were included in the study as per the inclusion and exclusion criteria.

Patient demographics

Age Distribution

**Figure 1:**
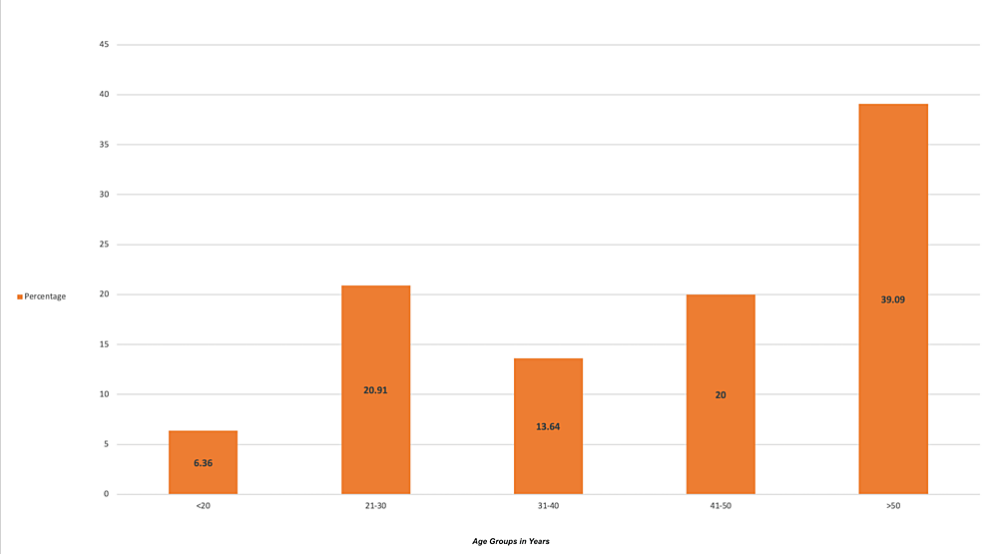
Bar graph showing age distribution in percentage Age group in years along the x-axis Percentage along the y-axis

In our study, seven (6.36%) patients were less than 20 years of age, 23 patients (20.91%) of patients were between 21 and 30 years, 15 (13.64%) patients were between 31 and 40 years of age, 22 (20%) were between 41 and 50 and 43 (39.09%) patients were >50 years. The mean age was 44±15 years. The patients in the study group ranged from 18 to 67 years. Figure 1 is a bar graph illustrating age distribution in percentage.

*Gender Distribution*

In this present study, 97% of patients were males and another 3% of patients were females. Male: Female ratio was 32:1.

Occupation

**Figure 2:**
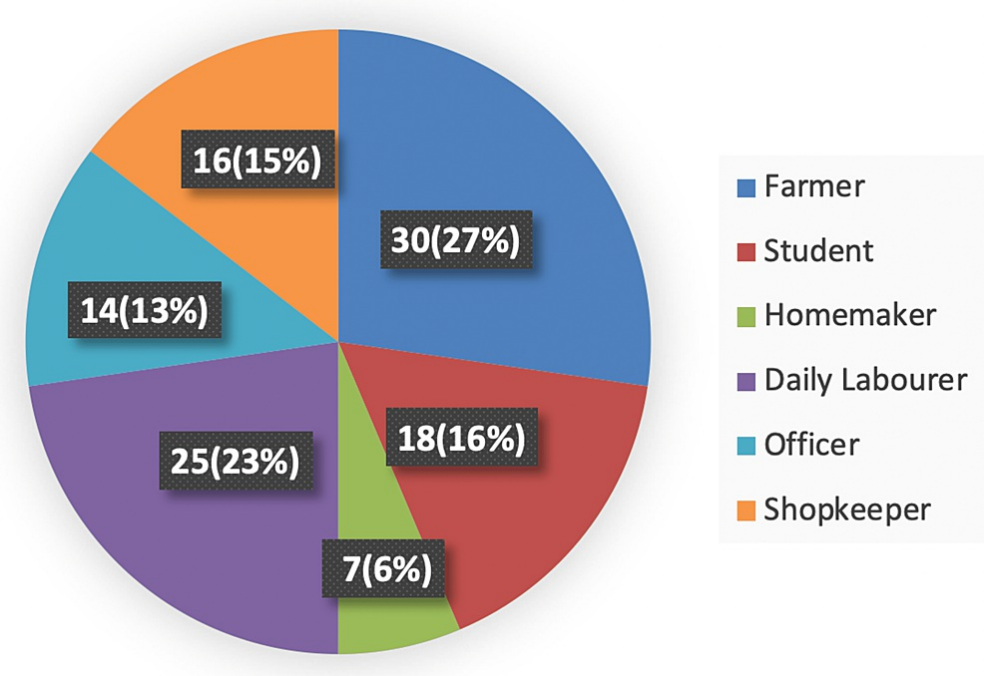
Pie chart showing the occupation of study participants in number and percentage

In this study, 27% were farmers by occupation, 16% were students, 6% were homemakers, 23% were labourers, 13% were officers and 16% were shopkeepers/vendors. Figure 2 is a pie chart showing the occupation-wise distribution of patients in number and percentage.

Risk Factors

The most common cause for the presence of hernia was lifting heavy objects in 55 (50%) and altered bowel movements; most of them had constipation, seen in 40 (36.36%) of the patients. Forty-two (38.1%) had diabetes, and 45 (40.9%) had respiratory problems, mainly chronic obstructive pulmonary disease. Twelve (10.9%) of the patients were alcoholics, and 52 (47.27%) of them were smokers.

Clinical Profile

In this current study, swelling was the clinical presentation in all patients. Groin pain with swelling was seen in 35% of patients. The distribution of patients with pain associated with swelling was not significant as tested using the Pearson Chi-Square test (P value: 0.554). Table 1 shows the clinical presentation of the inguinal hernia.

**Table 1:** Clinical presentation of inguinal hernia

Symptoms	Number of Patients	Percentage
Swelling	110	100
Pain with swelling	39	35.45

Pre-Operative Pain VAS Score

Pre-operative pain VAS (visual analog scale) score was mild in 51% of patients and moderate in 49% of patients with pain. The distribution of patients with pain associated with swelling was not significant as tested using the Pearson Chi-Square test (P value: 0.554).

Table 2 shows the number and percentage of patients in each grade of pre-operative pain score VAS.

**Table 2:** Pre-operative pain score

Pre-operative Pain Score VAS (Visual Analog Scale)	Number of Patients	Percentage
Mild	20	51.28%
Moderate	19	48.71%
Total	39	100%

Hernia Characteristics

In this series, the most common side of hernia was on the right side in 69 patients (63%), on the left, in 37 patients (33%) and bilateral in four (3%) patients.

Type of Hernia

The most common type of hernia was indirect hernia in 66 (60%) patients, followed by a direct hernia in 33 (30%) and both in 11 (10%). Table 3 shows the number of patients and their percentage for each type of hernia.

**Table 3:** Type of hernia number of patients and percentage

Type of Hernia	Number of Patients
Indirect	66 (60%)
Direct	33 (30%)
Both	11 (10%)
Total	110

## Discussion

The study was observational and conducted at a tertiary care centre in northern India; 110 patients were included in the study as per inclusion and exclusion criteria. After obtaining informed consent from all the participants, their clinical and demographic details were obtained.

In our study, we had 23 (20.9%) patients between 21 and 30 years, 22 (20.00%) patients between 41 and 50 years and 43 (39%) were >50 years. The mean age was 44±15 years. This was concordant with a study by de Goede B, wherein age around and above 50 was the most affected age group [6]. Similar results were also concluded by the study conducted by Ruhl CE, where the hernia was more common in ageing men of the range 40-59 years [7]. Also, similar results were shown by Sayanna and Basu [8,9], where the hernia was more common in older people for more than 50 years.

In the present study, 107 patients (97.27%) were males, and three patients (2.72%) were females. Male: Female ratio was 32:1. The preponderance of males to females was also seen in other studies, such as Burcharth J, in their study observed that inguinal hernias were 90.2% males and 9.8% females, which matches the results of the present study [10]. Ruhl et al. also reported similar findings [7]. Lau H et al. also reported that males are prone to have hernias [11].

The pathophysiology of hernia is based on the concept of increased abdominal pressure (mechanical effect) affecting a weak abdominal wall [12]. The main risk factor in the present study was found to be lifting heavy weights and strenuous work in 55%, followed by altered bowel habits in 36.36%. Smoking and diabetes were also associated risk factors with the hernia. Similarly, in their study, Sharma concluded that 52.4% of patients had hernias due to lifting heavy objects [12]. Also, in studies conducted by Constance Erin and Kumar, a similar association of risk factors was observed [7,13,14].

The most common type of hernia in this study was indirect in 60% of patients, followed by a direct hernia in 30%, and 10% of patients had both. Also, in this study, the most common side of hernia was on the right side, which was 63% on the right; on the left, it was 33% and bilateral in 4% of patients. This was also seen in a study by Nordback, where out of 469 patients, the right-sided were 207, the left-sided 146 were left-sided, and 116 were bilateral [15]. Similar was the case in the study by Gulzar et al., where out of 100 patients, 64 had right-sided inguinal hernia [16]. Garba ES from Nigeria conducted a survey and concluded that right inguinal hernias were commoner than left, with a ratio of 1.7:1.

In the present study, the swelling was the most common clinical presentation. Of 110 patients, 100 presented to the surgical clinic with groin swelling. This concurs with studies by Jenkins JT, where it was observed that groin swelling was the most common clinical presentation [17].

Groin pain with swelling was seen in 35% of patients. Among all patients having pain with the swelling, the pain was mild in 51% and moderate in 49% of patients. Similar observations were made by Page B et al. and others who observed mild to moderate pain in patients with inguinal hernia [18-20].

In our study, the most commonly involved patients were farmers by occupation, so farmers were 27%; followed by labourers at 23%; followed by students at 16%; shopkeepers/vendors at 16%; officers at 13%; and homemakers at 6%. It was observed that the preponderance of males was due to their involvement in more strenuous exercises, lifting weights and anatomical differences between the two. Also, the same results were published by Rao G in 2015, where heavy weight lifting was the commonest risk factor among male fishermen [20,21].

Limitations of the study can be ascribed to the limited sample size, which is insufficient to reflect a true picture of the disease. Hence, more multicentric studies with large sample sizes correlating aetiological or risk factors are recommended. Also, these type of studies needs to be conducted in different geographical areas so that they can be helpful for future studies in the prediction of the prevalence of inguinal hernias.

## Conclusions

Inguinal hernia is one of the commonest conditions seen in general surgery clinics among the male elderly group of patients. Commonly affected persons were farmers or labourers by occupation, as heavy weight lifting and strenuous exercises are among the most frequently involved risk factors for inguinal hernia. Altered bowel movement (constipation), respiratory disease (chronic obstructive airway disease) and smoking were also attributed as risk factors. Inguinal swelling is the most common mode of clinical presentation. Groin pain with swelling was seen in some of the patients. This pain was mild to moderate on VAS, and very few patients needed analgesics. Indirect type of hernia was more common than direct one. The right-sided hernia was more common than the left side, and the bilateral inguinal hernia was the least frequent. Definitive management of inguinal hernia is surgery.
